# Intracerebral Solitary Fibrous Tumor in Collision With Metastatic Colonic Adenocarcinoma

**DOI:** 10.7759/cureus.73851

**Published:** 2024-11-17

**Authors:** Brian H Le, Sandhya Konar, Robert M Barr, Gregory J Imbarrato

**Affiliations:** 1 Pathology, Novant Health Presbyterian Medical Center, Charlotte, USA; 2 Neurosurgery, Novant Health Presbyterian Medical Center, Charlotte, USA; 3 Radiology, Novant Health Presbyterian Medical Center, Charlotte, USA

**Keywords:** brain metastasis, collision tumor, dural-based tumor, intracranial solitary fibrous tumor, metastatic colorectal cancer

## Abstract

A collision tumor is a rare neoplastic lesion consisting of two or more coexisting, distinct cell line entities. In this report, we present the case of a 56-year-old male patient with a history of colon cancer who presented to the emergency room with visual deficits that had started about eight months earlier. An ophthalmologic examination reported left homonymous hemianopsia, prompting a brain MRI, which showed a right posterior temporal extra-axial mass concerning intracerebral metastatic colon cancer, in consideration of patient history. A right parietal craniotomy was performed, achieving gross total safe resection. The patient's preoperative left homonymous hemianopsia persisted, but no new neurological deficits were reported postoperatively. Pathologic examination revealed a solitary fibrous tumor in collision with metastatic colonic adenocarcinoma. The patient's family opted for comfort-directed care, given the patient's poor prognosis.

## Introduction

When encountering a solitary dural-based intracranial lesion on imaging or in pathologic tissue, the differential diagnosis is wide and includes primary neoplasms such as meningioma in its many variants and varying grades [1] and solitary fibrous tumor (SFT) [2]. Metastatic tumor is also highly considered in the differential diagnosis, especially in patients with a current or previous history of malignancy elsewhere [3]. Metastasis is especially favored when multiple intracranial space-occupying lesions are found, raising the possibility of dissemination to the leptomeninges [4]. Whether one or multiple lesions are identified, metastatic malignancy is typically favored as the top differential diagnosis in patients with known metastasis elsewhere in the body; as such, it would serve as a unifying diagnosis correlating with the global clinical scenario.

In the differential diagnosis of a solitary dural-based tumor, the possibility of a collision tumor is seldom considered. Collision tumors occur when more than one distinct neoplasm coexists as a single space-occupying lesion [5,6]. In the central nervous system (CNS), examples of collision tumors that have been described include a glioblastoma coexisting with a meningioma [5] and a meningioma colliding with an SFT [6], serving as evidence that two simultaneous neoplastic processes may occur coincidentally and simultaneously in the same location.

We present a case of an SFT and a metastatic colonic adenocarcinoma occurring as a dural-based collision tumor. In consideration of the patient's history of colon cancer with metastasis to the lung and liver, metastatic malignancy was initially considered. However, on histologic examination, there was morphologic evidence of two distinct neoplastic entities; these two neoplasms further demonstrated distinct immunophenotypes, thus facilitating the conclusion that two tumors may coexist or collide at a common location.

## Case presentation

**Figure 1:**
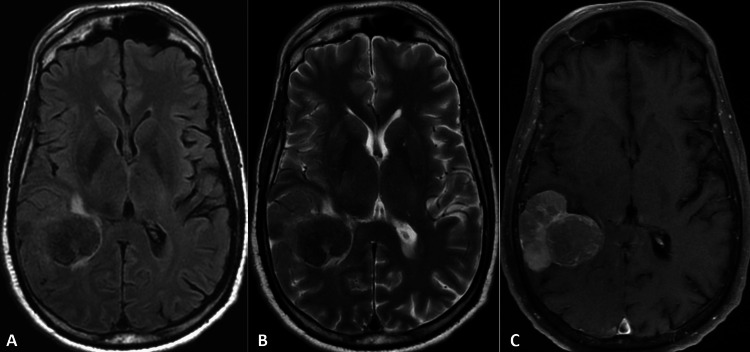
3T MRI demonstrates a heterogeneously enhancing lobulated extra-axial mass with minimal adjacent vasogenic edema, prominent flow voids, and decreased T2 signal. (A) FLAIR, (B) T2-weighted, and (C) postcontrast T1-weighted MRI: magnetic resonance imaging; FLAIR: fluid-attenuated inversion recovery

A 56-year-old male patient presented to the emergency room with complaints of visual changes. Ophthalmic examination was remarkable for a left homonymous hemianopsia. He had a past medical history that was significant for colon cancer with metastasis to the liver and lungs, for which he was receiving chemotherapy upon presentation. Additional medical history was notable for gastroesophageal reflux disease, diabetes mellitus type II, anemia, hypertension, and hyperlipidemia. Following ophthalmologic consultation, magnetic resonance imaging was performed and demonstrated a large dural-based mass (Figure 1). Metastatic disease was strongly considered in light of the underlying history. Several features were atypical for epithelial metastasis, however, including the low signal on T2 sequences, relative lack of vasogenic edema, and prominent flow voids. In addition, the lesion was fairly uniform and well-circumscribed despite its large size.

**Figure 2:**
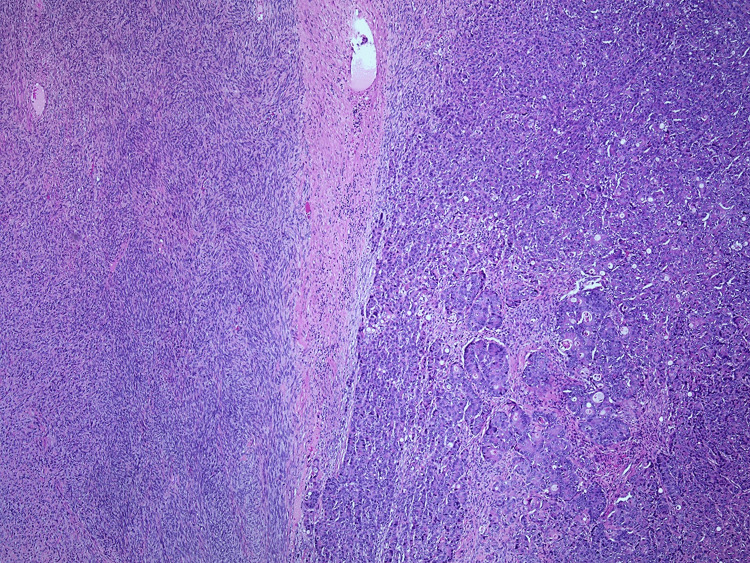
H&E-stained section (40x original magnification) demonstrating a proliferation of spindle cellular elements (left) at an abrupt interface with a complex glandular proliferation (right) H&E: hematoxylin and eosin

**Figure 3:**
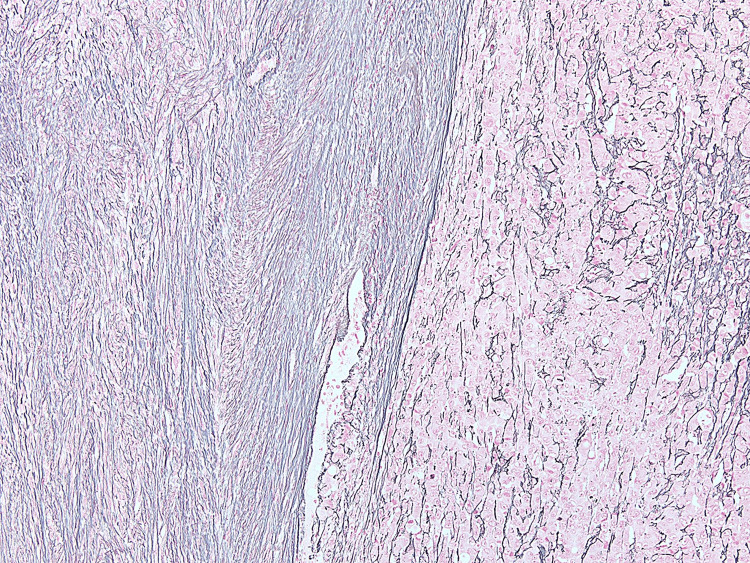
Reticulin stain (40x original magnification) accentuating pericellular fiber deposition within the spindle cell component (left), while highlighting cellular clusters in the glandular proliferation (right)

The patient's chemotherapy was held for a week, after which a right parietal craniotomy was performed. The specimen submitted for pathologic examination consisted of a 6.5 cm fragment of tan, rubbery tissue with attached dura; cut sections are tan-yellow with a rubbery consistency. Histologic sections showed a proliferation of complex, cribriform, and anastomosing glandular elements at the interface with a spindle cell proliferation (Figure 2) in a heterogeneous mix. The reticulin stain (Figure 3) accentuates pericellular fibrosis in the spindle proliferation while highlighting cellular clusters in the glandular proliferation.

**Figure 4:**
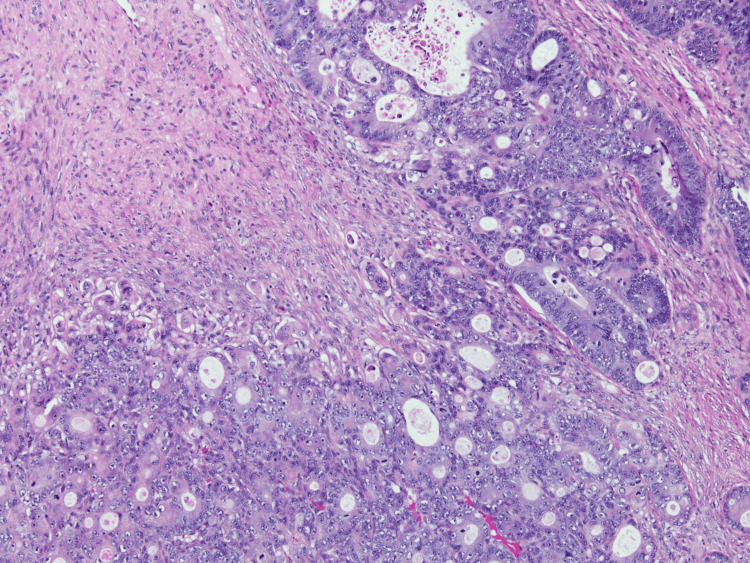
Glandular proliferation showing moderately pleomorphic cells with a cribriform architecture and prominent nucleoli (H&E stain, 100× original magnification) H&E: hematoxylin and eosin

**Figure 5:**
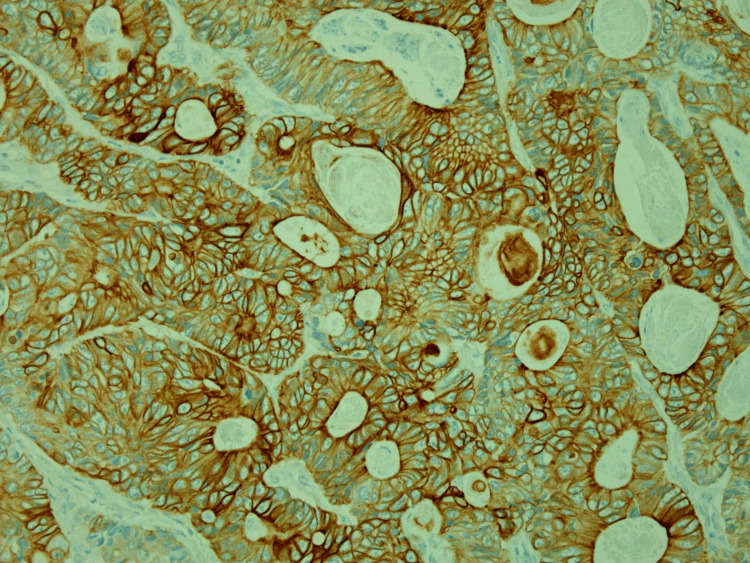
Cytokeratin 20 immunohistochemistry showing cytoplasmic reactivity in the glandular component (200× original magnification)

**Figure 6:**
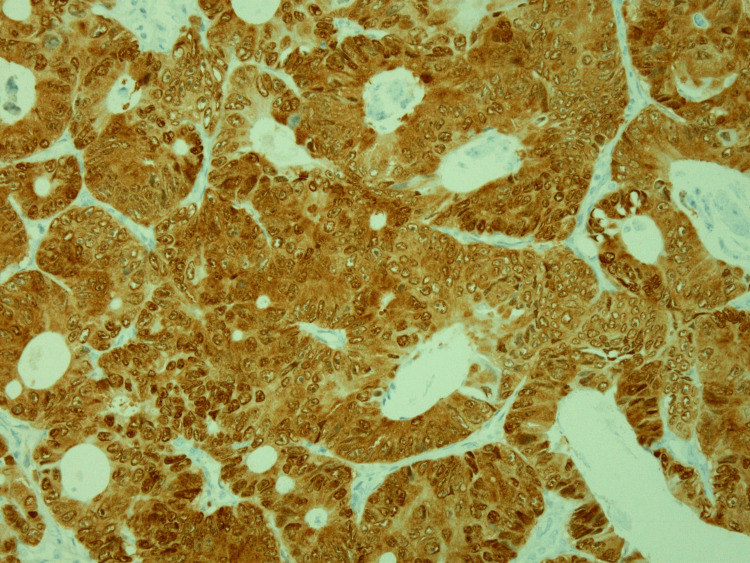
CDX2 immunohistochemistry showing diffuse nuclear reactivity in the glandular component (200x original magnification)

Within the glandular proliferation, neoplastic cells show moderate cellular pleomorphism with a cribriform architectural configuration (Figure 4). By immunohistochemistry, this neoplastic component shows diffuse cytoplasmic reactivity for cytokeratin 20 (Figure 5) and nuclear reactivity for CDX2 (Figure 6). The morphologic features of this epithelial glandular component, in consideration of immunophenotype, are entirely compatible with metastatic adenocarcinoma of colorectal origin, correlating with the patient's history of colon cancer with metastasis to the lungs and liver.

**Figure 7:**
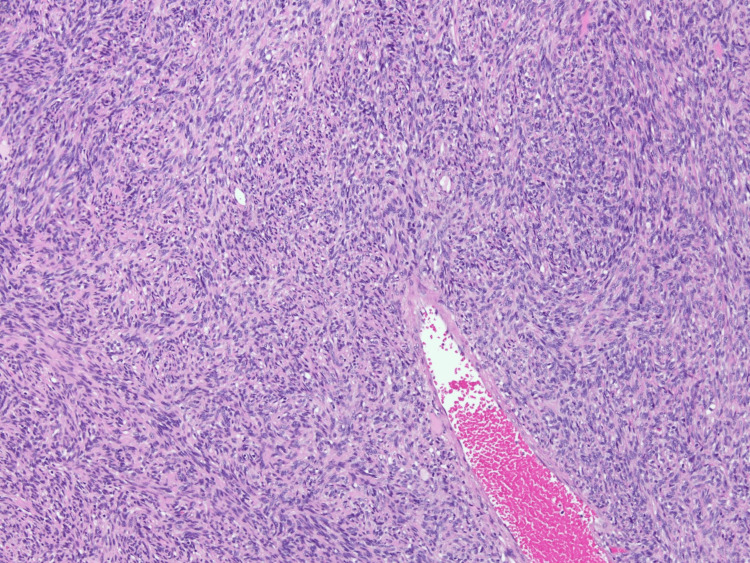
The spindle cell proliferation showing monomorphic cells in a fascicular architectural configuration (H&E stain, 100× original magnification) H&E: hematoxylin and eosin

Meanwhile, the distinct spindle cell proliferation shows a fascicular architectural arrangement of monomorphic cells (Figure 7), with a mitotic index of approximately 2 per 10 high-power fields. No regions of hemorrhage or necrosis are identified. Key histologic differential diagnoses for a tumor with these morphologic features include a meningioma, in particular, the fibroblastic variant, versus an SFT. Additional immunohistochemistry was performed to resolve this differential diagnosis, with appropriate controls verified.

**Figure 8:**
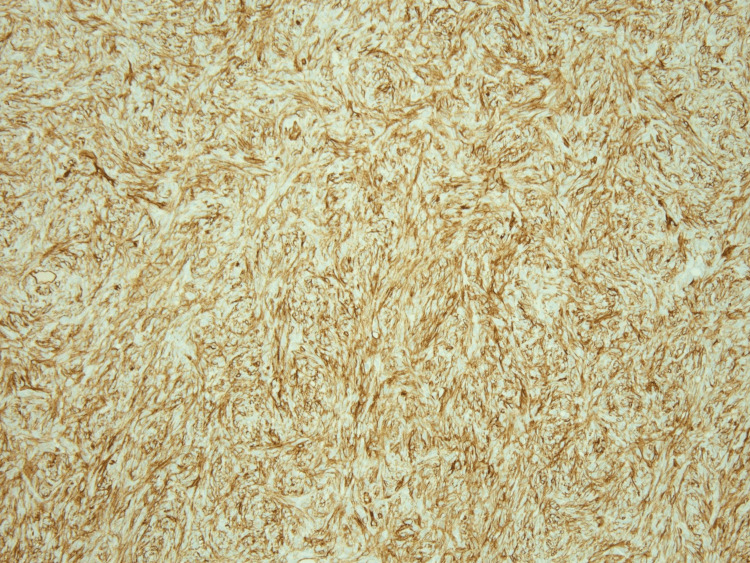
CD34 immunohistochemistry showing diffuse reactivity in the spindle cell proliferation (200× original magnification)

**Figure 9:**
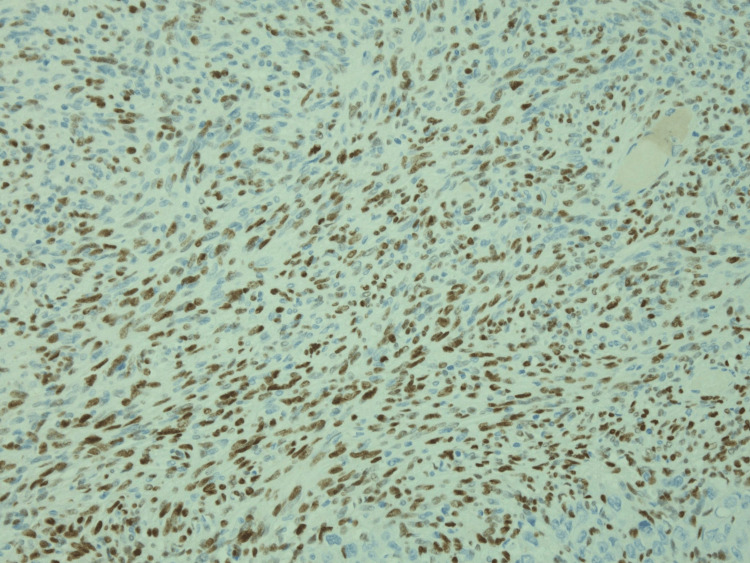
STAT6 immunohistochemistry demonstrating nuclear reactivity in the spindle cell proliferation (200x original magnification) STAT6: signal transducer and activator of transcription 6

These neoplastic cells show cytoplasmic reactivity for CD34 (Figure 8) and nuclear reactivity for signal transducer and activator of transcription 6 (Figure 9). Immunohistochemistry for the epithelial membrane antigen (EMA) is negative. The morphologic features, in consideration of this immunophenotype, are consistent with SFT, CNS World Health Organization (WHO) grade 1, based on the most current WHO classification.

In the presence of these two distinct neoplastic proliferations, the lesion reflects a collision tumor between a primary dural-based lesion (SFT) and metastatic adenocarcinoma of colonic origin.

Postoperatively, the patient's left homonymous hemianopsia persisted. However, there were no new neurological deficits. Due to the poor prognosis, driven in particular by the metastatic colonic adenocarcinoma, the patient's family decided to not pursue further aggressive treatments, opting for comfort care with hospice.

## Discussion

Key differential diagnoses for a solitary dural-based tumor include meningioma, SFT, and solitary metastasis [1-3]. It is rare to encounter two distinct lesions as part of a single or contiguous tumor focus [5,6].

SFT was previously recognized as a distinct pathologic entity from hemangiopericytoma when occurring in the CNS. Recently, the World Health Organization reevaluated these tumors, and a new classification scheme emerged, recognizing tumors previously falling into these diagnostic entities simply as "SFT” [3].

SFTs are spindle-cell neoplasms that originate from mesenchymal cells. These tumors often follow a benign course [7], often being confused with a diagnosis of meningioma, in particular fibroblastic meningioma. By histologic examination, SFTs show significant morphologic overlap with the fibroblastic variant of meningioma, as both tumors are characterized by bland-appearing spindle cells. Immunohistochemistry can help resolve this differential diagnosis, with fibroblastic meningioma typically expressing EMA, while SFT is reactive for CD34 and STAT6 [2,6]. In this case, reactivity for both CD34 and STAT6 was consistent with SFT, while negativity for EMA helped to exclude fibroblastic meningioma.

Dissemination of colon cancer to the brain is a rare phenomenon, with an incidence rate of only approximately 2% [8]. Nevertheless, in this patient with a history of colonic metastasis to other distant sites, intracranial metastasis was of top consideration in the radiographic differential diagnosis.

In this patient, the prognosis was deemed to be driven primarily by the metastatic colonic adenocarcinoma rather than by the SFT component. Given the overall poor prognosis, the family chose the pathway of comfort-directed care, and the patient died within a month of resection.

## Conclusions

The occurrence of SFT and metastatic adenocarcinoma of the colon as a collision tumor in the CNS is an unusual phenomenon. Patient history and body imaging findings significantly influence prioritization of differential diagnostic possibilities when considering a dural-based lesion. This case demonstrates several important points when considering the differential diagnosis of a solitary, dural-based intracranial tumor.

In the presence of a history of known malignancy, metastasis should lead the list of differential diagnostic possibilities. In the absence of known malignancy or suspected malignancy by body imaging, a primary dural-based neoplasm should lead to a differential diagnosis. This would include meningioma and its variants, as well as SFT. Regardless of the scenario, however, at the time of histologic examination, the possibility of more than one tumor type should be considered, especially when there is morphologic heterogeneity within the lesion.

This case illustrates that, while rare, metastatic colonic adenocarcinoma may occur simultaneously with a primary SFT. This serves to raise awareness that consideration should be given to the possibility of collision tumors.
